# Human Lymph Node-Derived Fibroblastic and Double-Negative Reticular Cells Alter Their Chemokines and Cytokines Expression Profile Following Inflammatory Stimuli

**DOI:** 10.3389/fimmu.2017.00141

**Published:** 2017-02-14

**Authors:** Patricia Severino, Diana Torres Palomino, Heliene Alvarenga, Camila Bononi Almeida, Denise Cunha Pasqualim, Adriano Cury, Paolo Rogério Salvalaggio, Antonio Luiz De Vasconcelos Macedo, Maria Claudina Andrade, Thiago Aloia, Silvio Bromberg, Luiz Vicente Rizzo, Fernanda Agostini Rocha, Luciana C. Marti

**Affiliations:** ^1^Albert Einstein Research and Education Institute, Hospital Israelita Albert Einstein, São Paulo, Brazil; ^2^Programa de Alergia e Imunopatologia, Faculdade de Medicina da Universidade de São Paulo (FMUSP), São Paulo, Brazil; ^3^Hospital Israelita Albert Einstein, São Paulo, Brazil; ^4^Endocrinology Department, Santa Casa de Misericórdia de Sao Paulo, São Paulo, Brazil

**Keywords:** human lymph nodes, fibroblastic reticular cell, double-negative cells, podoplanin, chemokines

## Abstract

Lymph node (LN) is a secondary lymphoid organ with highly organized and compartmentalized structure. LNs harbor B, T, and other cells among fibroblastic reticular cells (FRCs). FRCs are characterized by both podoplanin (PDPN/gp38) expression and by the lack of CD31 expression. FRCs are involved in several immune response processes but mechanisms underlying their function are still under investigation. Double-negative cells (DNCs), another cell population within LNs, are even less understood. They do not express PDPN or CD31, their localization within the LN is unknown, and their phenotype and function remain to be elucidated. This study evaluates the gene expression and cytokines and chemokines profile of human LN-derived FRCs and DNCs during homeostasis and following inflammatory stimuli. Cytokines and chemokines secreted by human FRCs and DNCs partially diverged from those identified in murine models that used similar stimulation. Cytokine and chemokine secretion and their receptors expression levels differed between stimulated DNCs and FRCs, with FRCs expressing a broader range of chemokines. Additionally, dendritic cells demonstrated increased migration toward FRCs, possibly due to chemokine-induced chemotaxis since migration was significantly decreased upon neutralization of secreted CCL2 and CCL20. Our study contributes to the understanding of the biology and functions of FRCs and DNCs and, accordingly, of the mechanisms involving them in immune cells activation and migration.

## Introduction

The immunological function of lymph nodes (LNs) includes the initiation of antigen-specific immune responses as well as the induction of tolerance when required. These secondary lymphoid organs possess a structured morphology and harbor a variety of cell populations, including lymphocytes B, T, and other less characterized cell populations. Among these, the fibroblastic reticular cells (FRCs) are phenotypically characterized by both podoplanin expression (PDPN, gp38) and by the absence of CD31 expression (1–3). FRCs are known to participate in several immune response processes, including immune cells homing and activation, but essential mechanisms fundamental to these functions are still under investigation (2, 4). The double-negative cells (DNCs) constitute a poorly understood cell population within LNs. These cells do not express gp38 or CD31, their localization within LNs is unclear, and their function remains to be elucidated (5–7).

Fibroblastic reticular cells and DNCs share stromal cells phenotype and are known to secrete chemokines (8). They are involved in the control of migratory patterns and positioning of immune cells during homeostasis or inflammatory response (8). In animal models, FRCs and DNCs react to inflammatory stimuli such as interleukin 1 (IL-1), interferon (IFN)-α, -β, and -γ, and tumor necrosis factor (TNF)-α, by altering chemokines gene and protein expression profiles (9–12). Chemokines constitute a large family of small cytokines involved in immune cells chemotaxis. Some chemokines are considered pro-inflammatory and released upon infection, while others are considered homeostatic and involved in the control of cell migration during tissue development or maintenance. The physiologic importance of this family of mediators results from their specificity because its members induce recruitment of well-defined leukocyte subsets (13, 14).

Chemokines are secreted by FRCs within LNs where they orchestrate the encounter between diverse immune cell types. In murine LNs, FRCs are localized in the paracortex and they produce CCL19 and CCL21, attracting T and dendritic cells *via* CCR7 (5). IL-7 secretion is also attributed to FRCs (2). IL-7 contributes to the maintenance of naïve T cells survival and IL-7 signaling is regulated by IL-7Rα, a specific receptor, in FRCs (2, 15). Other studies in murine models and humans reported FRCs also producing IL-6 and IL-15 (5, 8, 16).

Our study evaluates the expression of interleukins and chemokines in human-derived FRCs and DNCs under standard culture conditions and following inflammatory stimuli using IFN-γ and TNF-α + IL-1β treatment. IFN-γ induces lymphocytes differentiation and is produced by several immune cell subtypes in response to inflammatory stimulation (17). In contrast, the cytokine TNF-α is secreted by T and B cells in response to antigenic stimulation (18), while IL-1β takes part in several immunologic processes, including T cells differentiation into Th1 and Th2 (10).

We demonstrate that, following inflammatory stimulation, the secretion of interleukins and chemokines by human LN-derived FRCs and DNCs partially differed from those identified in murine models. In addition, FRCs showed a broader range of chemokines being upregulated when compared with DNCs. These results suggest that distinct immune cells subsets may interact with either FRCs or DNCs. Accordingly, dendritic cells demonstrated an increased migration potential toward FRCs following treatment with TNF-α and IL-1β and their migration was significantly decreased upon neutralization of secreted CCL2 or CCL20. These results imply functional differences between FRCs and DNCs within LNs.

## Materials and Methods

### Human LNs

Peripheral or mesenteric human LNs were obtained from four individuals with different ages and clinical status (larynx cancer—LN03, diverticulitis—LN12, breast cancer—LN15 patients, and an organ donor for liver transplantation—LN16) submitted to surgical procedures. All research participants signed an informed consent (approved by Hospital Israelita Albert Einstein research ethics committee under CAAE number: 07768712.4.0000.0071). All LNs were submitted to histopathological examination, which confirmed that they were not visually affected by the disease.

### Histopathology and Immunohistochemistry

Formalin-fixed paraffin-embedded LN samples were sectioned into 3 µm thick slices and transferred to microscope slides. The slides containing the tissue were stained with hematoxylin–eosin (HE). Immunohistochemical staining was performed using Autostainer DAKO automated immunohistochemistry slide processing platform according to the manufacturer’s instructions and individual staining was performed for CD20 (clone: L26), CD3 (Polyclonal), and CD68 (clone: PGM-1) antibodies, all from Dako (Glostrup, Denmark). Histopathology and immunohistochemistry were performed by the Clinical Pathology Laboratory from Hospital Israelita Albert Einstein.

### Enzymatic Digestion of LNs

For the isolation of the stromal cells, the LN was shredded with a scalpel. Lymphocytes released in the media were collected and frozen for further analysis and the remaining of the lymphoid tissue was washed with Hank’s balanced salt solution (HBSS, Gibco, Carlsbad, CA, USA) and incubated at 37°C for 30 min in 9 mL of HBSS, 1 mL of Accutase (Innovative Cell Technologies, San Diego, CA, USA), 0.6 ng/mL of collagenase type II, and 1 µL of turbo DNAse (Invitrogen, Carlsbad, CA, USA). The tube containing the tissue underwent mechanical stirring every 5 min during the 30-min incubation period. After incubation, the LN fragments were vigorously stirred with a needle to complete the tissue disruption. Samples were then centrifuged (500 *g*, 5 min, 22°C), and the pelleted cells were suspended in DMEM-LG and cultured (5% CO_2_, 37°C) in 25 cm^2^ culture flasks. After the third passage, the plastic adherent cells were phenotypically characterized and sorted in two cell populations, FRCs and DNCs, according to the procedures described in the following section.

### FRCs and DNCs Immunophenotyping and Sorting by Flow Cytometry

Immunophenotyping of cells was performed with the following antibodies: CD34-PE (clone: 8G12) from BD Biosciences, San Jose, CA, USA; CD14-Alexa 700 (clone: M5E2), CD29-APC (clone: MAR04), CD35-FITC (clone: E11), CD44-PerCP-Cy5.5 (clone: G44-26), CD73-PE (clone: AD2), CD90-PE-Cy7 (clone: 5E10), HLA-DR-APC-H7 (clone: G46-6), CD106-FITC (clone: 51-10C9) all from BD Pharmingen, San Diego, CA, USA; CD31-V450 (clone: WM59), CD45-V500 (clone: H130), CD105-PE-CF546 (clone: 266) all from BD Horizon, San Jose, CA, USA, and gp38/PDPN-APC (polyclonal) from R&D Systems, Minneapolis, MN, USA, and fluorescence minus one (FMO) was used as a fluorescence background control. Briefly, 1 × 10^5^ cells were incubated in the dark/room temperature for 30 min in the presence of antibodies at concentrations recommended by the manufacturers. Cells were then washed with buffered solution and at least 10,000 events were acquired using FACS LSRII FORTESSA (BD Biosciences). Cell populations were characterized according to the following reference markers: FRCs: gp38+, CD31−; DNCs: gp38−, CD31−; lymphatic endothelial cells: gp38+, CD31+; blood endothelial cells: gp38−, CD31+ and/or CD106+; follicular dendritic cells (FDCs): gp38+, CD35+; hematopoietic cells: CD45+, CD14+ or CD34+; stromal cells markers: CD29, CD44, CD73, CD90, and CD105. Data were analyzed using the FACSDIVA (BD Biosciences) and/or the FlowJo software (Tree Star, Ashland, OR, USA).

After characterization, cells were sorted according to gp38/PDPN expression: gp38/PDPN positive, the FRCs, and gp38/PDPN negative, the DNCs, using FACSARIA II cell sorting (BD Biosciences). Cell sorting was performed in order to achieve high cell population purity (>95%). After cell sorting and expansion in culture, an additional step of gp38/PDPN expression verification was performed before every experiment; if purity was below 95% cells were resorted, as described in Figure S1 in Supplementary Material.

### Differentiation of Cells Into Adipocyte-, Chondrocyte-, and Osteocyte-Like Cells

In order to evaluate FRCs and DNCs stromal cell characteristics, we studied their differentiation potential. Both cell subtypes were cultured in specific differentiation media for 21 days following literature guidelines (19, 20). For adipogenic differentiation, the medium used was Alpha-MEM (Gibco) supplemented with 10% FBS (Gibco), 1 µM dexamethasone, 100 µg/mL 3-isobutyl-1-methylxanthine IBMX, 10 µg/mL insulin, and 100 µM indomethacin (all from Sigma, St. Louis, MO, USA). The osteogenic differentiation medium used was Alpha-MEM supplemented with 10% FBS, 1 µm dexamethasone, 2 µg/mL ascorbic acid, and 10 µM β-glycerophosphate (all from Sigma St. Louis, MO, USA). For chondrogenic lineage differentiation, we used the medium Alpha-MEM supplemented with 10% FBS, 1 µm dexamethasone, 2 µg/mL ascorbic acid and 6.25 µg/mL insulin from Sigma, and 10 ng/mL TGF-β from R&D Systems. After 21 days, cells were fixed and stained with specific dyes to confirm differentiation: the adipogenic cultures were fixed in 4% paraformaldehyde and stained with oil red (Sigma) to indicate the lipid droplets formation inside the cells; cells in osteogenic culture were fixed in 50% ethanol at 4°C and stained with Alizarin Red (Sigma) to indicate the calcium deposition in the culture, and the chondrogenic cultures were fixed in crescent ethanol concentrations (70, 90, and 100%) and stained with toluidine blue (Sigma) to identify the proteoglycan-rich matrix.

### Dendritic Cells Differentiation and Maturation

Monocytes were derived from peripheral blood obtained from four healthy volunteers after they signed an informed consent. Peripheral blood cells were diluted 1:3 with phosphate-buffered saline. The suspension was transferred to a 15-mL conical tube containing 5 mL of Ficoll-Paque 1.077 density (GE Healthcare, UK) and centrifuged (30 min/500 *g*) at 22°C. The cells from the interface were collected, ressuspended, and centrifuged again (5 min/500 *g*). Cells were then separated according to CD14 expression using a magnetic selection column (Miltenyi Biotec, Bergisch Gladbach, Germany). The CD14 cell population was dispensed into six-well plates containing X-vivo 15 medium (Cambrex) supplemented with antibiotic-antimycotic solution (Gibco). To generate immature dendritic cells (iDCs), the cells were cultured in the presence of human recombinant IL-4 (20 ng/mL) and GM-CSF (50 ng/mL) (both from R&D Systems, Minneapolis, MN, USA) for 6 days (20). Mature DCs were obtained after iDC stimulation with LPS 100 ng/mL for 24 h (Sigma-Aldrich). Monocytes and monocyte-derived immature and mature DCs were analyzed for chemokines receptor as described in the following section.

### Lymphocytes Isolation

Lymphocytes were isolated from peripheral blood obtained from 11 healthy volunteers after they signed an informed consent. An aliquot of whole blood cells was immediately stained for lymphocyte-specific markers and chemokines receptors as described in the following section. Lymphocytes were also isolated from the LNs as described in the Section “Enzymatic Digestion of LNs.” LN-derived lymphocytes (*n* = 6) were also stained for lymphocyte-specific markers and chemokines receptors as described in the following section. The chemokine receptors expression of the peripheral blood lymphocytes and the LN-derived lymphocytes were compared.

### Dendritic Cells and Lymphocytes Chemokine Receptors Detection by Flow Cytometry

Immunophenotyping of lymphocytes, monocytes, and monocyte-derived DCs was performed with the following antibodies: CD3-PerCP-Cy5.5 (clone: SK7), CD33-APC (clone: P67.6), and HLA-DR-PerCP-Cy5.5 (clone: G46.6), from BD Biosciences, CD209-FITC (clone: DCN46), CD8-APC-Cy7 (clone: SK1), CD80-PE (clone: L307.4), CD86-PE (clone: 2331-FUN-1), CCR7-FITC (clone: 3D12), CD14-PerCP-Cy5.5 (clone: M5E2), CD4-Alexa-700 (clone: RPA-T4), CXCR4-PE (clone: 12G5), CCR2-Alexa-647 (clone: 48607), CCR6-PE-Cy7 (clone: 11A9) from BD Pharmingen, CCR3-BV421 (clone: 5E8), CCR5-PE-CF594 (clone: 2D7/CCR5) from BD Horizon. FMO was used as a fluorescence background control. Briefly, 1 × 10^5^ cells were incubated in the dark/room temperature for 30 min in the presence of antibodies at concentrations recommended by the manufacturers. We used FACS Lysing solution (BD Biosciences) for red cells lysing after staining of whole blood. Cells were then washed with buffered solution and at least 10,000 events were acquired using FACS LSRII FORTESSA (BD Biosciences).

Data were analyzed using the FACSDIVA (BD Biosciences) and/or the FlowJo software (Tree Star, Ashland, OR, USA). The gating strategy is described in Figure S2 in Supplementary Material.

### ELISA

The FRCs and DNCs were cultured with or without inflammatory stimuli provided by either TNFα (25 ng/mL) in combination with IL-1β (5 ng/mL) treatment or IFN-γ (50 ng/mL) treatment (21, 22). The culture supernatant was removed 24 h after treatment and frozen for ELISA assays. The cells that remained attached to the culture plate were harvested for RNA extraction (see RNA Extraction). We used ELISA Duo set (R&D Systems) for quantification of CCL2, CCL19, and CCL20, Quantikine (R&D Systems) for IL7 and CXCL12 quantification. For IL-6, IL-8, and IL-10, we used reagents from Immunotools (Immunotools, Germany). All procedures followed the manufacturers’ instructions. Plate readings were carried out using the microplate reader SpectraMax^®^ i3 (Molecular Devices, Sunnyvale, CA, USA). Readings were performed at 450/540 nm and the standard curve was set at 4 parameters logistic or log/log curve fit, according to each manufacturer’s protocol.

### Migration Assay

The FRCs and DNCs were cultured with or without inflammatory stimuli provided by either IFN-γ (50 ng/mL) or TNF-α (25 ng/mL) in combination with IL-1β (5 ng/mL) treatments. Monocyte-derived iDCs were stained with 5 µM/mL of carboxyfluorescein succinimidyl-ester (CFSE-Invitrogen) for detection using green fluorescence, as previously described (23). The iDCs (2 × 10^4^ cells) were placed in 3.0 µm transwell inserts (Anopore membranes, Nunc Kamstrup, Denmark) within chambers in 24-well plates containing FRCs or DNCs previously treated for 24 h with inflammatory signals, or in plates with untreated FRCs and DNCs. After 24 h in culture, the insert was removed from the culture plate and cells resting in the upper part of the insert (lose cells) were removed. The filter was removed from the chambers and the fluorescent cells trapped in the transwell were counted using a fluorescence microscope (Olympus IX51).

Aiming to evaluate the impact of CCL2 and CCL20 on dendritic cells migration, iDCs (2 × 10^3^ cells) were placed on 8.0 µm transwell inserts within chambers (Translucent Membranes, Greiner Bio-One, Vienna, Austria) in 24-well plates containing either FRCs or DNCs previously treated or untreated for 24 h with inflammatory signals. Cells were treated with neutralizing antibodies for CCL2 (2 µg/mL) or CCL20 (5 µg/mL) (ELISA capture antibodies, R&D Systems). After 24 h, the insert was removed from the culture plate and the cells that transmigrated toward FRCs or DNCs were visualized in the lower part of the chamber and counted using the detection of green fluorescence by a fluorescence microscopy (Olympus IX51).

### RNA Extraction

RNA extraction was carried out using RNAeasy Kit (Qiagen, Valencia, CA, USA) according to the manufacturer’s instructions. For RNA quantification and quality analysis, we used the NanoVue spectrophotometer (GE Healthcare Life Sciences, Little Chalfont, UK).

### Gene Expression Analysis Using DNA Microarrays

To understand patterns of cytokine and chemokine gene expression by FRCs and DNCs, we used DNA microarrays. We compared FRCs and DNCs that were treated or not with IFN-γ (50 ng/mL) or TNF-α (25 ng/mL) in combination with IL-1β (5 ng/mL). Cells from four LNs were used for this assay: two LNs isolated from cancer patients (LN04 and LN15), one from a diverticulitis patient (LN12), and one isolated from a healthy liver donor (LN16). Gene expression levels were evaluated using the DNA microarray SurePrint G3 Human Gene Expression 8 × 60K v2 Microarray Kit (Agilent Technologies, USA). Labeling of probes with cyanine 3 dye (Cy3), hybridization, and washing procedures strictly followed the manufacturer’s protocol (One Color Quick Amp Labeling Kit, Agilent Technologies). The arrays were scanned using a SureScan Microarray Scanner (Agilent Technologies, USA) using default parameters for Agilent microarrays. Initial data processing was performed using the Agilent Feature Extraction software (version 10.5), and GeneSpring (Agilent Technologies, USA) was used for data normalization (quantile normalization) and transformation. For the differential gene expression analysis, the gene-level normalized intensities were compared between sample pairs and a fold-change was calculated for each cell type per LN (treated vs untreated cells). The expression data are deposited at the Gene Expression Omnibus site (accession number GSE89612 associated with platform GPL16699).

### Statistical Analysis

The statistical analysis was performed using the GraphPad Prism program (version 6.02). The Student’s *t*-test was used for comparison between two groups or ANOVA for multiple comparisons with Bonferroni correction, when necessary. A minimum *p* < 0.05 significance was considered for all experiments.

## Results

### Pathological Analysis of LNs Evidentiate Tissue Integrity

The pathological analysis showed tissue integrity and absence of metastasis in the cancer-derived LNs. LNs were also evaluated for cells distribution using histochemistry markers for CD3, CD20, and CD68 (24) and, accordingly, we observed that T lymphocytes were present in the paracortex region, B lymphocytes were within the follicles, and the macrophages were distributed throughout the LN. We observed that follicles in cancer-derived LNs were not as well delimited as those obtained from the healthy liver donor or from the diverticulitis patient (Figures S3A–C in Supplementary Material).

### Cell Subpopulations Isolated from LNs Were Classified as Stromal Cells

**Figure 1:**
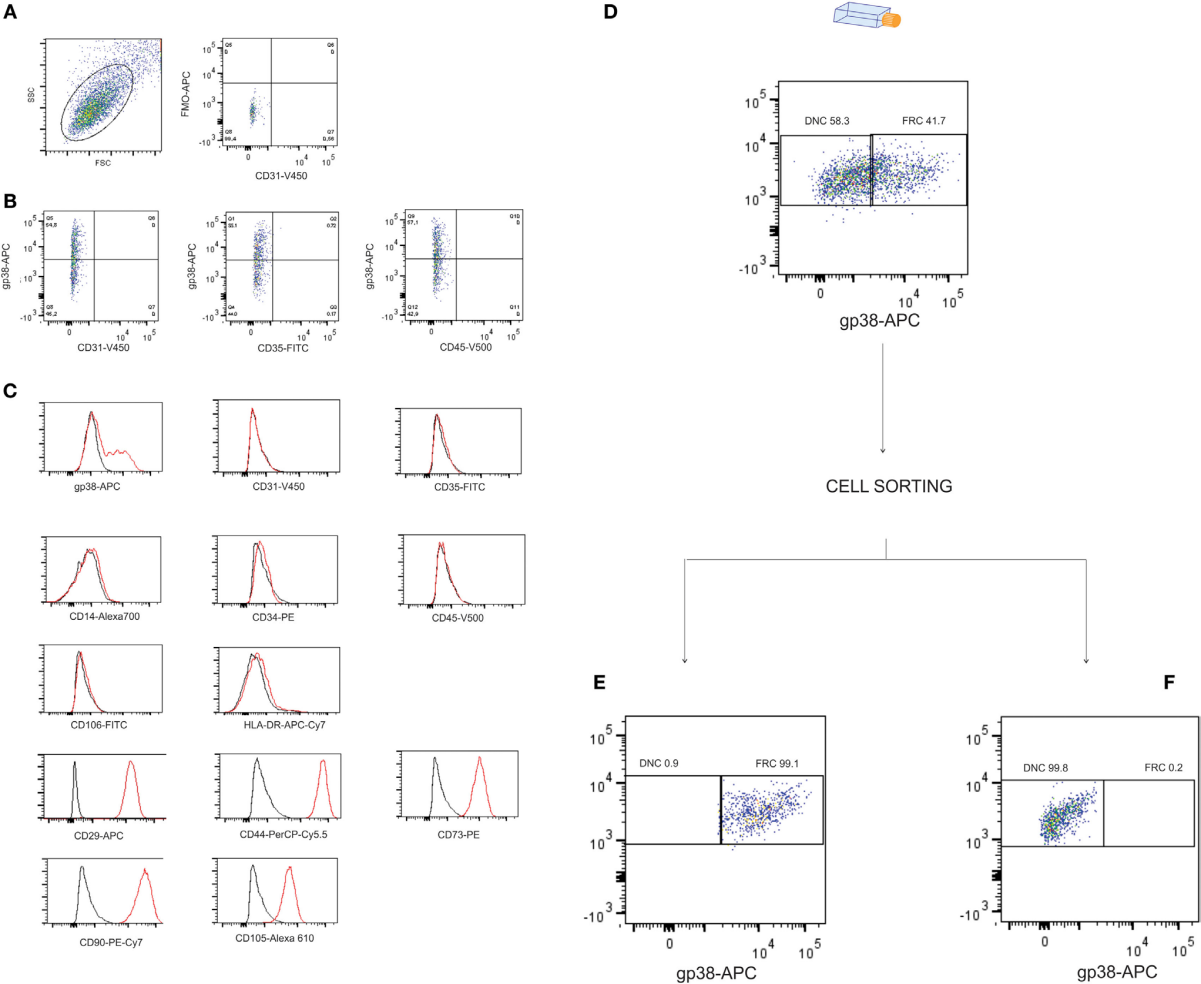
**Phenotypic profile and cell sorting of stromal cells isolated from human lymph nodes (LNs)**. Stromal cells isolated from human LNs (four samples) were analyzed by flow cytometry for: **(A)** size vs granularity and fluorescence minus one (FMO) for gp38 (FMO vs CD31), histograms of gated cells show unlabeled cells (black line) and stained cells (red line), **(B)** LN stromal cells were phenotyped in fibroblastic reticular cells (FRCs) or double-negative cells (DNCs) by gp38 and blood endothelial cells, lymphatic endothelial cells, and follicular dendritic cells were excluded due to absence of CD31, CD35, CD106, and HLA-DR expression, **(C)** FRCs and DNCs showed positivity for CD29, CD44, CD73, CD90, CD105 and no expression of CD14, CD34, CD45, indicating absence of hematopoietic cells, **(D)** flow cytometry data showing purity over 97% in the cell sorting of **(E)** FRCs and **(F)** DNCs. All four LNs were fully characterized; this figure displays the results from LN12.

**Figure 2:**
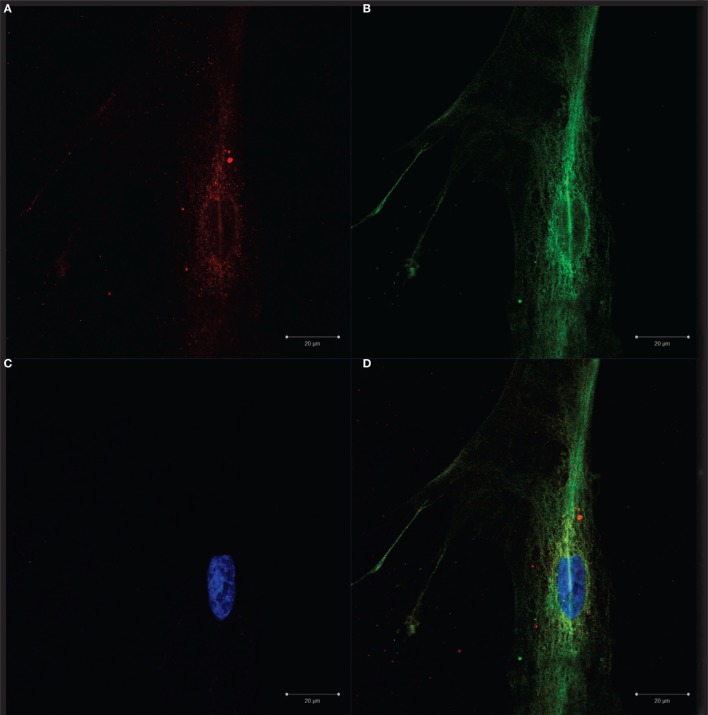

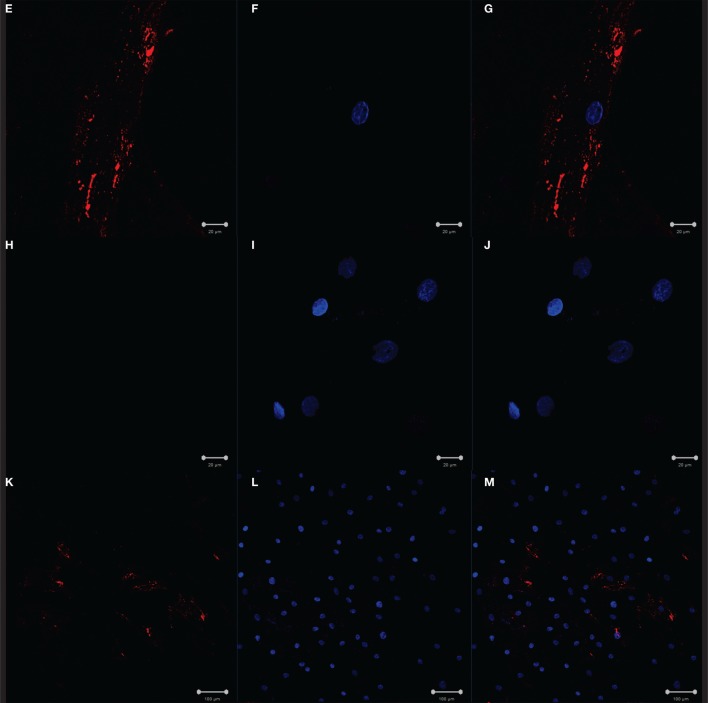

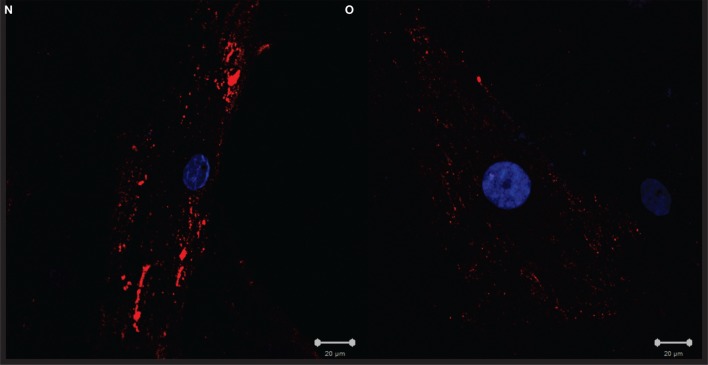
**Confocal microscopy**. Confocal microscopy revealing the morphology of fibroblastic reticular cells (FRCs) isolated and expanded *in vitro*: **(A)** intracellular gp38/PDPN staining (red), **(B)** actin microfilament stained with tubulin (green), **(C)** nuclei detection with DAPI (blue), **(D)** merge (red, green and blue), **(E,K)** cell membrane staining with gp38/PDPN (red), **(H)** control (secondary antibody only) displaying absence of unspecific staining in red, **(F,I,L)** nuclei detection with DAPI (blue), **(G,J,M,N,O)** merge (red and blue), **(M,N,O)** images of mixed culture with FRCs and double-negative cells.

**Figure 3:**
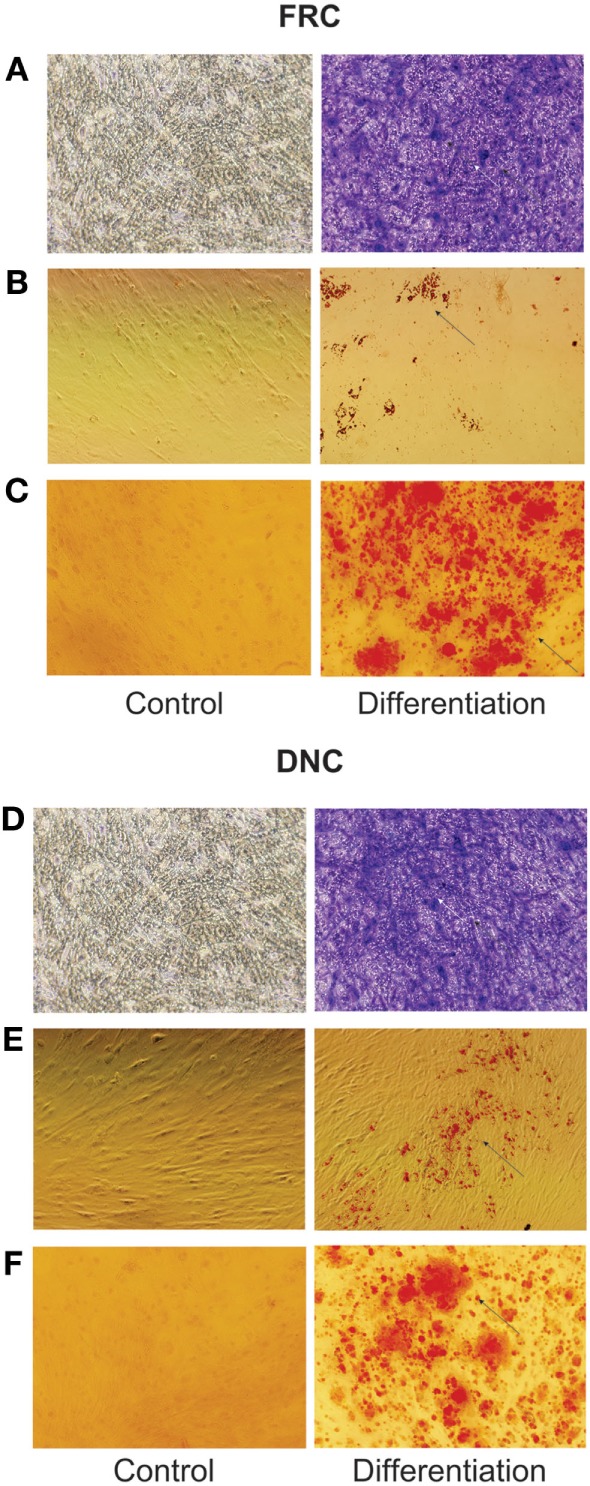
**Fibroblastic reticular cells (FRCs) and double-negative cells (DNCs) differentiation into three lineages**. FRCs and DNCs were separated by cell sorting for differentiation: **(A–C)** FRCs and, **(D–F)** DNCs; **(A,D)** FRCs and DNCs staining with toluidine blue showing formation of proteoglycans matrix on chondrocyte differentiation, **(B,E)** FRC and DNC displaying formation of cytoplasm lipid droplets, stained with oil red, indicating adipocyte differentiation, **(C,F)** FRCs and DNCs staining with Alizarin red indicates the formation of calcium matrix during osteocyte differentiation. Images at original magnification of 20×. All four LN were fully characterized (this figure displays the results from LN12).

Cells isolated from the human LNs and used in this study were uniform in size and granularity (FSC vs SSC). They did not express markers for endothelial cells (CD31 and CD106), and the absence of CD35 expression indicated the absence of FDCs among them (Figures 1A,B). These cells also did not express hematopoietic markers such as CD14, CD34, CD45, or HLA-DR, they expressed CD29, CD44, CD73, CD90, and CD105 (Figure 1C) and were classified into FRCs and DNCs subpopulations according to PDPN/gp38 expression (Figures 1D–F). They displayed fibroblastic shape and were plastic adherent (Figures 2A–O). FRCs and DNCs were able to differentiate into osteocyte-, chondrocyte-, and adipocyte-like cells (Figures 3A–F). Thus, we concluded that, as expected, the human FRCs and DNCs displayed stromal cells characteristics that fulfill the international phenotypic criteria determined by International Society for Cellular Therapy (19).

### Cytokine and Chemokine Expression Was Induced by Inflammatory Factors

Microarray gene expression analysis was used to evaluate the expression of interleukins and chemokines and respective receptors directly involved in immune cells activation and migration following inflammatory stimuli. We hypothesized that DNCs and FRCs are cell subpopulations that may respond differently to such conditions. We compared results from standard culture conditions and cells submitted to 24 h treatment with either IFN-γ or TNF-α + IL-β. A summary of the results is displayed in Table 1, and complete data reports are displayed in Tables S1–S4 in Supplementary Material.

**Table 1:** **Gene expression of chemokines and interleukins in double-negative cells (DNCs) and fibroblastic reticular cells (FRCs) following treatment with IFN-γ or TNF-α + IL-1β**.

	Chemokines and interleukins induced by IFN-γ		Chemokines and interleukins induced by TNF-α + IL-1β	
GENE	DNC	FRC	DNC	FRC
CCL2	12.65	937.80	26.74	3,097.21
CCL3	–	–	58.92	158.58
CCL5	15.93	–	99.25	53.83
CCL7	30.26	25.98	38.53	38.12
CCL8	109.60	398.84	43.45	–
CCL11	–	–	–	6.48
CCL13	72.59	141.17	–	7.83
CCL20	–	–	–	259.11
CXCL1	–	–	667.98	1,769.66
CXCL2	51.94	–	465.36	233.16
CXCL3	–	10.41	1,015.14	1,531.52
CXCL5	–	–	–	38.23
CXCL6	–	–	–	779.07
CXCL8	–	58.90	2,141.93	4,932.57
CXCL9	–	1,064.60	–	–
CXCL10	191.19	491.06	122.44	11.78
CXCL11	174.2	247.68	–	–
CCL3L3	–	–	–	10.01
IL-1α	–	–	–	127.54
IL-1β	–	–	579.39	1,023.30
IL-4I1	8.07	–	19.61	–
IL-6	–	–	26.28	219.28
IL-11	–	–	–	352.47
IL-15	–	334.28	–	29.85
IL-32	13.23	–	10.34	5.61
IL-33	6.26	20.37	22.45	189.35
IL-36γ	–	–	11.87	28.78
IL-18 binding protein	–	168.29	–	–
IL-1RN	–	–	14.71	405.44
IL-3RA	7.20	–	–	–
IL-7R	–	–	–	159.50
IL15RA	6.67	8.32	3.49	–

*Results represent the average fold-change between treated cells vs controls in four lymph nodes (LNs) and absent results indicate that uniform upregulation (fold-change > 2.0) was not observed in all four LNs. The DNCs and FRCs used in this experiment were derived from LN04, LN12, LN15, and LN16. Individual data of each LN was included in Tables S1–S4 in Supplementary Material*.

After IFN-γ treatment, we observed upregulation of chemokine genes expression in both FRCs and DNCs, but with marked differences (Table 1). CCL5 and CXCL2 were more expressed in DNCs than in FRCs, while CCL2, CCL8, CCL13, CXCL3, CXCL8, CXCL9, CXCL10, and CXCL11 were more expressed in FRCs (Table 1). Alterations in the expression levels were also observed after TNF-α + IL-1β treatment, with chemokines being more expressed in treated FRCs when compared to DNCs, and a group of chemokines upregulated only in treated FRCs (CCL3L3, CXCL2, and CXCL11).

**Figure 4:**
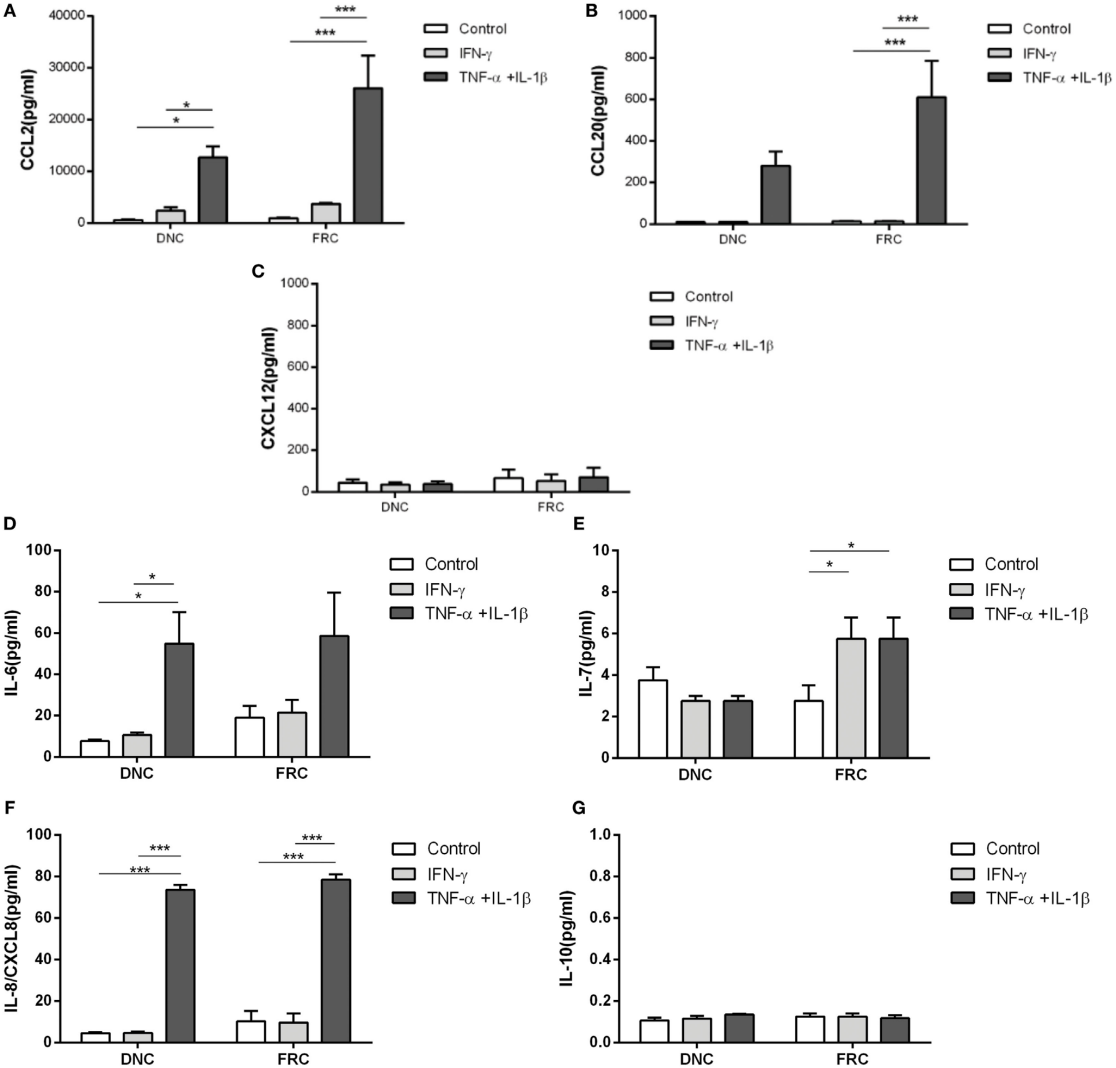
**Chemokine secretion by fibroblastic reticular cells (FRCs) and double-negative cells in homeostasis and under inflammatory stimuli**. Lymph node stromal cells (derived from LN04, LN12, LN15, and LN16) cultured during 7 days and treated with TNFα + IL-1β (light gray bar) or IFN-γ (dark gray bar) for 24 h. White bars correspond to cells cultured without treatment. Chemokines and cytokines were measured in each cells supernatant and results presented are the average and standard deviation, **(A)** CCL2, **(B)** CCL-20, **(C)** CXCL-12, **(D)** IL-6, **(E)** FRCs IL-7 [IL-7 assay range for cell culture supernatant should be between 0.2 and 10 pg/mL (sensitivity level is 0.1 pg/mL)], **(F)** IL-8/CXCL-8, **(G)** IL-10 secretion was lower than 0.1 pg/mL and was not altered by cytokines stimulation (*p < 0.05, ***p < 0.001).

To confirm that the differential patterns of chemokines and interleukins expression following treatment was dependent upon the cell type, CCL2, CCL20, CXCL8, and CXCL12 protein levels were evaluated in the culture medium using ELISA. TNFα + IL-1β stimulation of FRCs and DNCs significantly enhanced CCL2 secretion (26,050 ± 12,608 and 12,677 ± 2,178 pg/mL, respectively) when compared to the correspondent IFN-γ treatment (3,668 ± 524 and 2,390 ± 1,262 pg/mL) or correspondent untreated cells (913 ± 280 and 587 ± 141 pg/mL), corroborating the gene expression results (Figure 4A). Similarly, CCL20 secretion in FRCs culture was also significantly enhanced after TNFα + IL-1β stimulation (610 ± 176 pg/mL) when compared to IFN-γ (13 ± 2 pg/mL) or untreated cells (14 ± 3 pg/mL) (Figure 4B). There was a moderate secretion of CXCL12 in untreated DNCs (44 ± 33 pg/mL) and in FRCs (67 ± 81 pg/mL), which was not altered by inflammatory stimulation (Figure 4C), while CCL19 was undetectable in every condition.

IL-6 secretion was significantly enhanced in DNCs after TNFα + IL-1β stimulation (54.8 ± 15.2 pg/mL) when compared to IFN-γ (10.7 ± 1.2 pg/mL) and untreated cells (7.6 ± 0.9 pg/mL) (Figure 4D), but there were no significant differences in IL-6 secretion by FRCs (Figure 4D). However, we observed a variation in the expression levels of IL-6 among FRC samples and a relatively high secretion of IL-6 by untreated FRCs (18.21 ± 13 pg/mL) or IFN-γ stimulated FRCs (21.42 ± 12 pg/mL).

Enhanced IL-7 secretion by FRCs was observed after TNFα + IL-1β stimulation (5.7 ± 1.0 pg/mL) when compared to untreated cells (2.7 ± 0.7 pg/mL), but no differences were observed in DNCs (Figure 4E).

CXCL8 (IL-8) secretion was significantly enhanced in FRCs and DNCs after TNFα + IL-1β stimuli (78.4 ± 2.6 and 73.5 ± 2.5 pg/mL, respectively) compared to their respective untreated controls (10.2 ± 4.9 and 4.4 ± 0.4 pg/mL), while IFN-γ did not alter this secretion significantly (Figure 4F). IL-10 secretion by FRCs or DNCs was almost undetectable under standard and inflammatory conditions (Figure 4G).

Our results suggest that the chemokine and cytokine expression of these subpopulations in the LN microenvironment may be important to guarantee homeostasis, which is modified under inflammatory conditions, possibly leading to specific responses.

### Chemokine Receptors Expressed by Monocytes, Dendritic Cells, and Lymphocytes Suggest Differential Interaction between FRCs, DNCs, and Immune Cells

**Figure 5:**
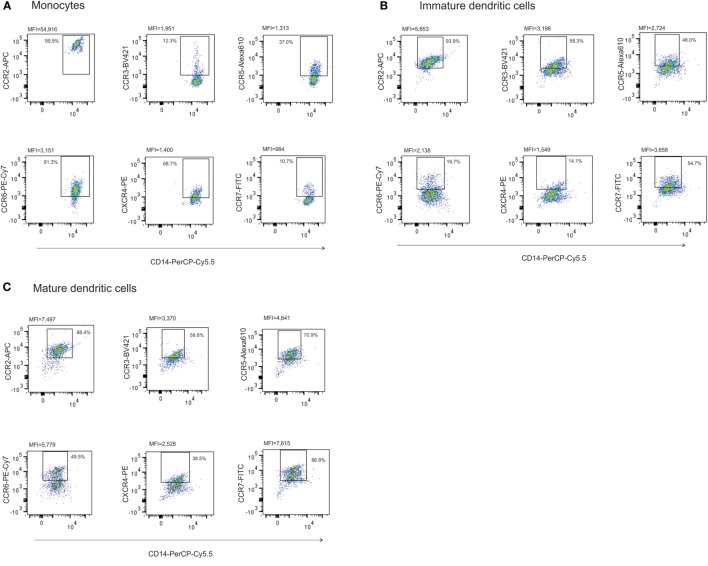

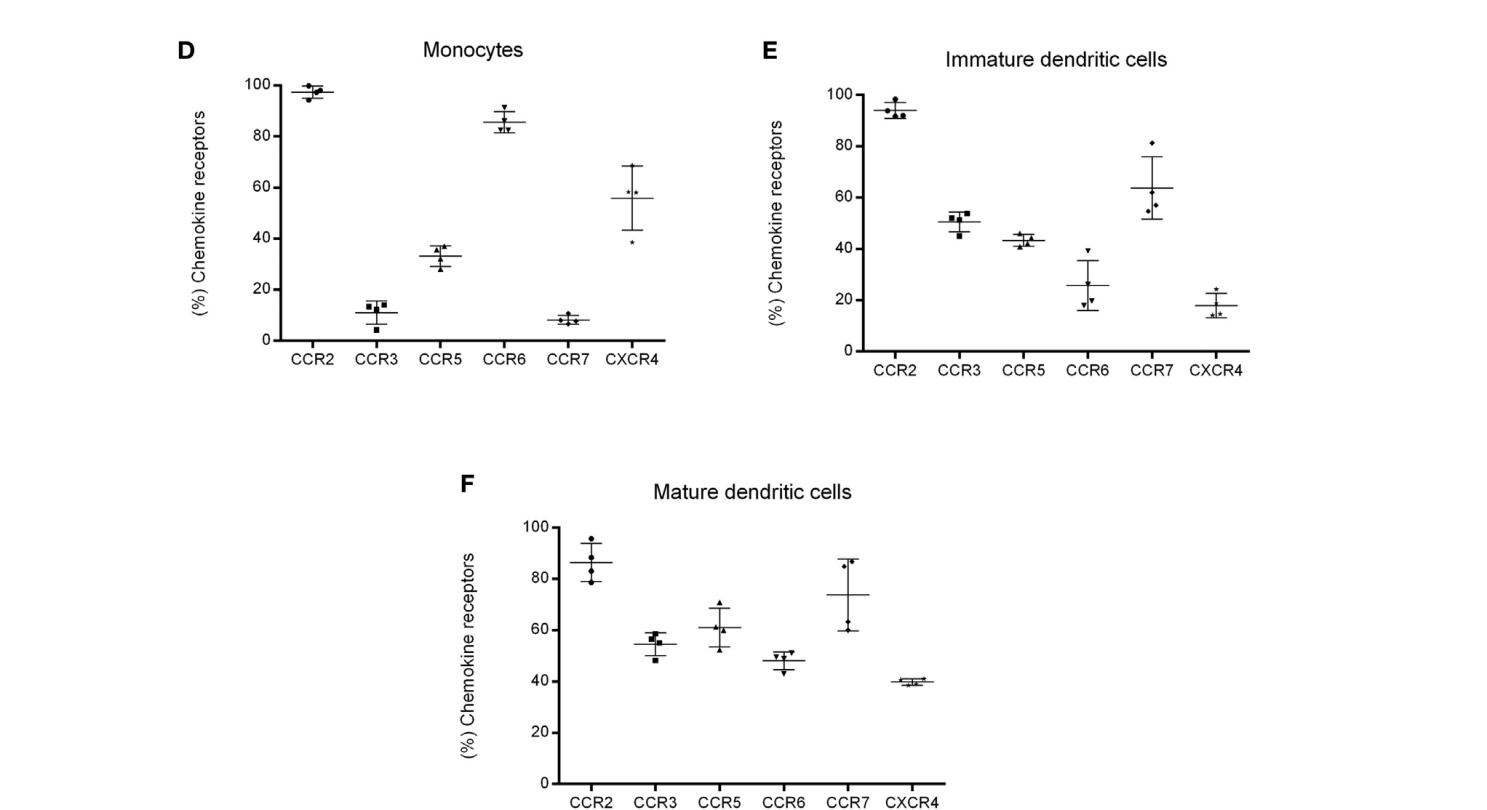
**Chemokine receptors on monocytes from peripheral blood and monocyte-derived DCs**. **(A)** Monocytes were isolated by positive selection (CD14) and cultured for 7 days with IL-4 (20 ng/mL) and GM-CSF (50 ng/mL) to differentiate into, **(B)** immature and, **(C)** mature dendritic cells when stimulated with LPS (100 ng/mL); each cellular subset was evaluated for CCR2, CCR3, CCR5, CCR6, CCR7, and CXCR4 expression by flow cytometry and the frequency of positive cells to chemokine receptors was plotted in the corresponding graphs to **(D)** monocytes, **(E)** immature and, **(F)** mature dendritic cells. Results are expressed in percentage and median of fluorescence intensity (MFI). Peripheral blood was collected from four healthy individuals for this assay.

**Figure 6:**
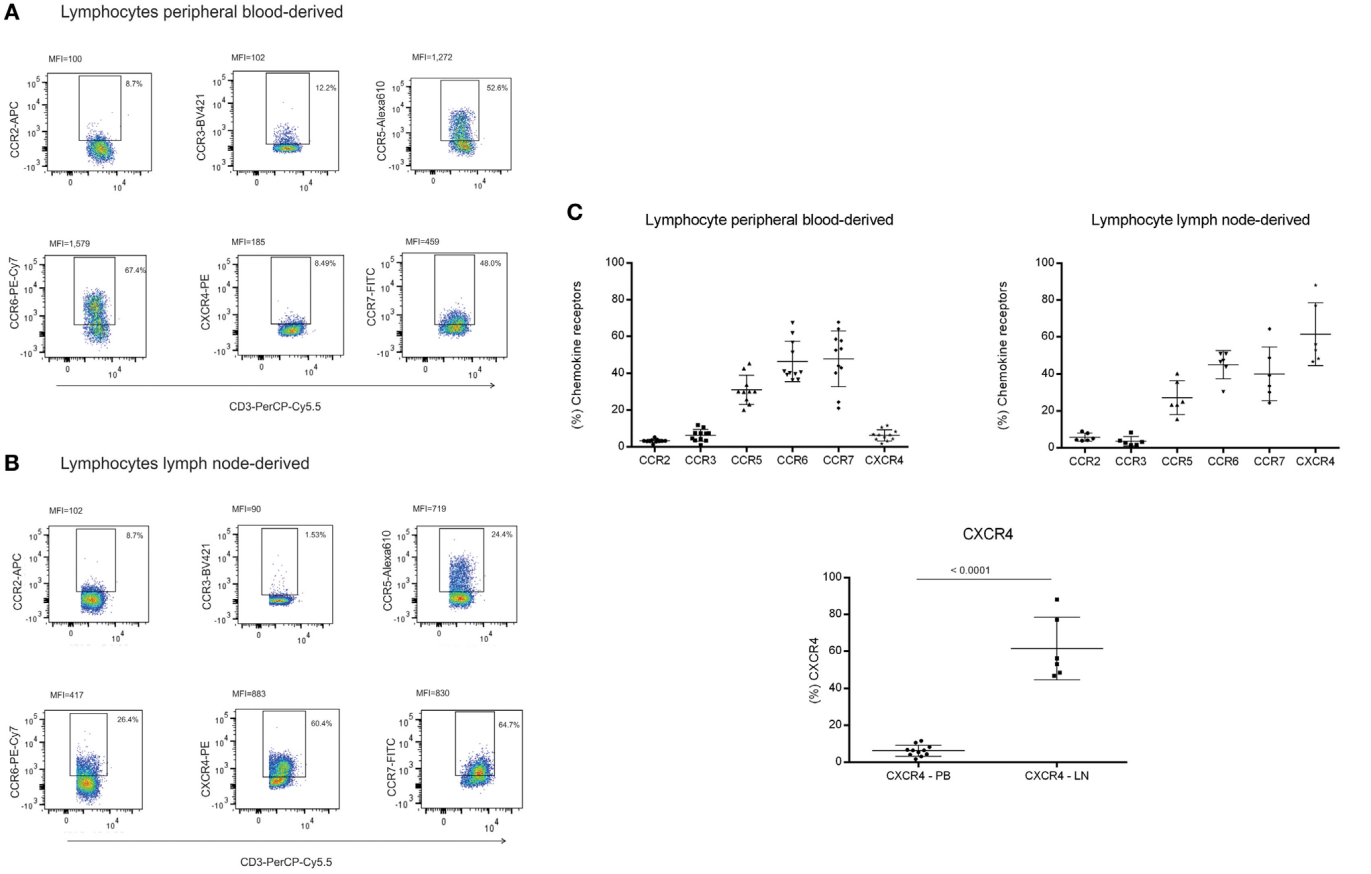
**Chemokine receptors in lymphocytes**. Lymphocytes derived from: **(A)** peripheral blood of healthy volunteers (*n* = 11) and **(B)** lymphocytes derived from lymph node (LN) (*n* = 06, LN04—larynx cancer; LN08, LN16, and LN24—liver donors; LN12—diverticulitis; LN15—breast cancer) were evaluated for CCR2, CCR3, CCR5, CCR6, CCR7, and CXCR4 expression by flow cytometry. Results are expressed in percentage and median of fluorescence intensity (MFI), **(C)** Comparative graphs for CXCR4 expression between peripheral and LN lymphocytes (*p* < 0.001).

Fibroblastic reticular cells and DNCs presented different expression levels of cytokines and chemokines upon inflammatory stimulation. In order to verify whether these expression patterns could lead to a differential interaction with immune cell populations, we evaluated the expression of chemokine receptors in lymphocytes, monocytes, and monocyte-derived DCs. CCR2 was highly expressed in monocytes and DCs, both immature and mature. There were variations in CCR6 and CXCR4 expression and increased expression of CCR3, CCR5, and CCR7 according to the maturation status of the DCs (Figures 5A–F). Regarding lymphocytes, we observed that there were subpopulations expressing all the chemokine receptors evaluated in this study. In addition to those receptors, lymphocytes isolated from LNs expressed higher CXCR4 levels compared to peripheral blood samples (Figures 6A–C). The chemokine receptors analyzed suggest that the interaction between FRCs and DNCs and immune cells may differ. For instance, we observed an intense upregulation of CCL2 and CCL20 genes and secretion by FRC when compared to DNCs and their respective receptors, CCR2 and CCR6, are expressed in iDCs.

### Monocyte-Derived Dendritic Cells Migrate toward TNF-α + IL-1β-Treated FRCs

**Figure 7:**
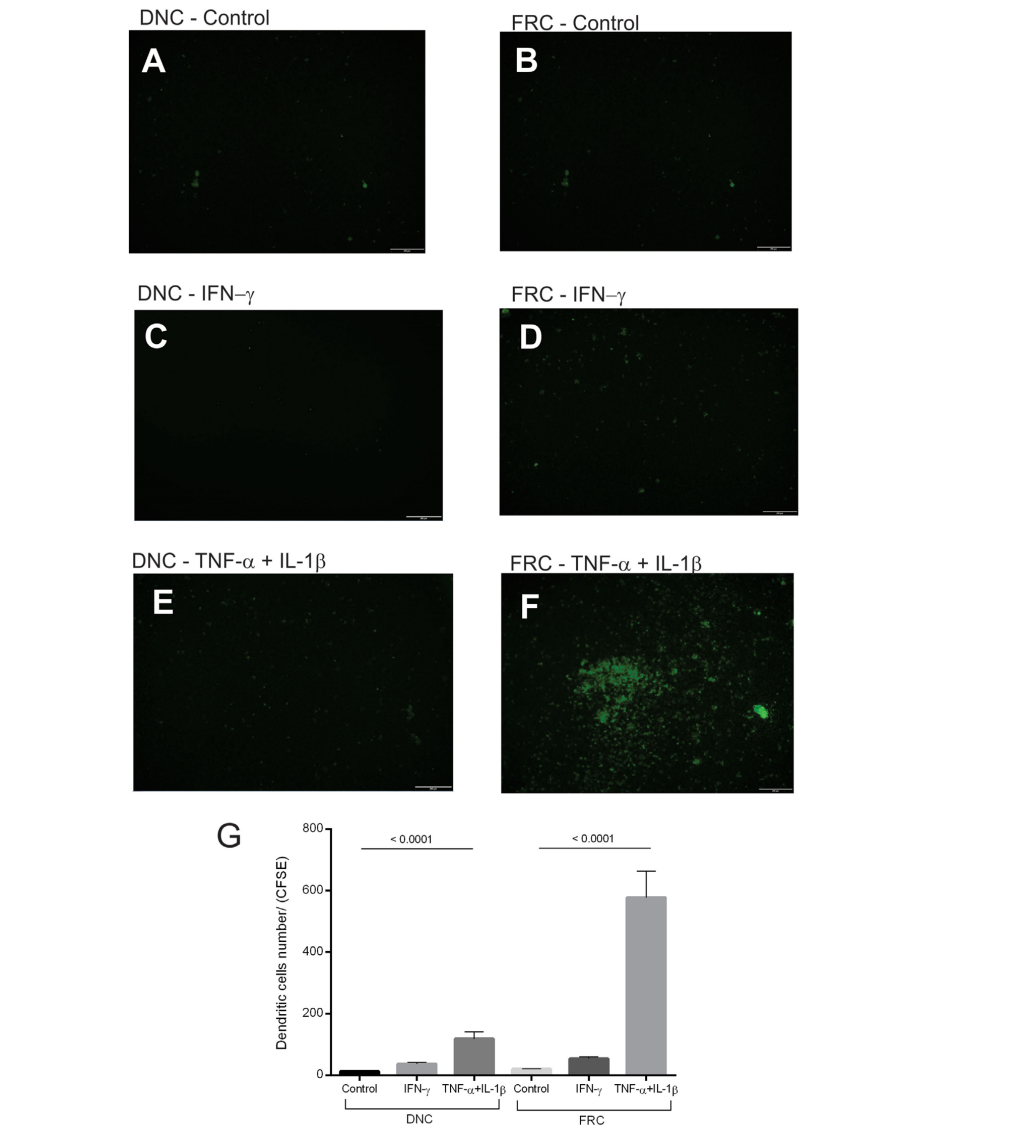

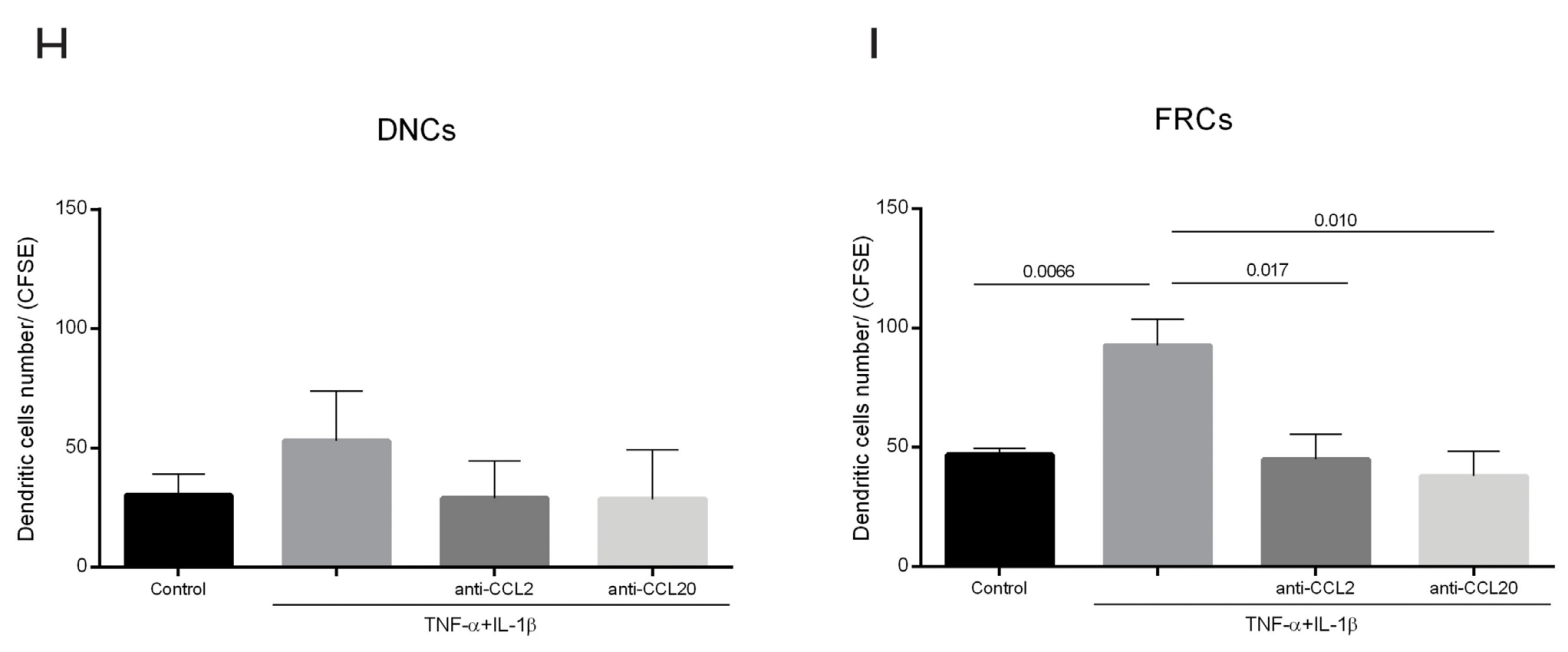
**Migration assay for immature dendritic cells (iDCs)**. Confluent double-negative cells (DNCs) or fibroblastic reticular cells (FRCs) were treated with IFN-γ (50 ng/mL) or TNF-α (25 ng/mL) + IL-1β (5 ng/mL) for 24 h. CFSE-labeled monocyte-derived iDCs were added into the upper chamber of the transwell in the absence or presence of neutralizing antibodies [CCL2 (2 µg/mL) or CCL20 (5 µg/mL)]. iDCs stained trapped in the transwell or that transmigrated were visualized by CFSE staining (green). **(A)** Control DNCs, **(B)** control FRCs; iDCs that migrated toward INF-γ treated (green), **(C)** DNCs or **(D)** FRCs; iDCs that migrated toward TNF-α + IL-1β treated (green) **(E)** DNCs or **(F)** FRCs, **(G)** quantification of iDCs trapped in the transwell membrane, **(H)** quantification of iDCs that transmigrated toward DNCs controls and treated with CCL2 and CCL20 neutralizing antibodies, **(I)** quantification of iDCs that transmigrate toward FRCs controls and treated with CCL2 and CCL20 neutralizing antibodies. The LN04 and LN16 were used for this assay (*n* = 3).

The effect of inflammatory stimulation of FRCs and DNCs on immune cell migration was evaluated by a migration assay using iDCs. We used monocyte-derived DCs and not LN isolated cells, due to the limited number of conventional DCs within LNs and/or circulating in peripheral blood (25). We found a more intense migration of iDCs toward TNF-α + IL-1β-treated FRCs, less migration toward similarly treated DNCs, and lower migration rates toward the untreated cells (Figures 7A–F). iDCs migration toward IFN-γ-treated FRCs or DNCs was low and similar to the controls (Figure 7G).

Since iDCs expressed CCR2 and CCR6 receptors and migrated toward TNF-α + IL-1β-treated FRCs that expressed and secreted high levels of CCL2 and CCL20, we used neutralizing antibodies for these chemokines to confirm their involvement in iDCs migration (Figures 7H–I). Our results showed that iDCs migration toward TNF-α + IL-1β stimulated FRCs or DNCs treated independently with anti-CCL2 or anti-CCL20 was reduced compared to the treated controls. However, these results were statistically significant only for FRCs, thus confirming the participation of FRCs in iDCs recruitment through CCR2/CCL2 and CCR6/CCL20 activation.

## Discussion

Stromal cells were first identified and characterized in murine LNs as structural cells (5, 26, 27). Recently, reports indicate that these cell populations are directly involved in the immune response initiation and regulation (16, 27). However, to our knowledge, there are only three recent articles that evaluated the potential immune functions of stromal cells from human LNs (10, 28, 29). None of these studies performed extensive *in vitro* studies with different stimulation as we described in this manuscript.

In one of these studies (10), the authors isolated and compared human dermal fibroblasts with LN reticular fibroblastic cells gene expression after treatment with TNF-α, IL-4, IL-6, and IL-13. However, in their study, there was no distinction between FRCs and DNCs subsets. A second study compared the differences in gene expression between FRCs isolated from murine skin draining LNs and mesenteric LNs in steady state. However, they only described in detail the successful isolation and culture methods of human FRCs (28). The third study evaluated the role of stromal cells from tonsils and bone marrow on B cells derived from follicular lymphoma, but there was no distinction between FRCs and DNCs subsets (29). In contrast, our study characterized plastic adherent cells derived from human LNs as stromal cells according to their phenotype and their ability to differentiate in three mesodermal lineage cells and we distinguished FRCs and DNCs among adherent cells based on gp38/PDPN and CD31 expression.

Literature reports show that murine FRCs and DNCs respond to inflammatory stimuli by expressing several molecules involved in the immune response (9–12). We chose to use IFN-γ and TNF-α + IL-1β as inflammatory stimuli based on their specific but also diverse mechanisms of inducing immune cell response (17, 18, 30).

Our results show that upon stimulation with either IFN-γ or TNF-α + IL-1β, the population of DNCs and FRCs derived from human LNs upregulated chemokines gene expression and protein levels. However, there were differences between treatments and cell subpopulation. For instance, CCL2 and CCL20 secretion was significantly increased in FRCs in response to TNF-α + IL-1β stimulation when compared to IFN-γ treatment, untreated controls or similarly treated DNCs, corroborating the higher gene upregulation in FRCs compared to DNCs.

CCL2, also known as monocyte chemoattractant protein-1, is involved in chemotaxis of CCR2-expressing memory T cells, monocytes, and DCs to sites of inflammation produced by either tissue injury or infection. CCL20 also known as macrophage inflammatory protein-3 α (MIP-3α) is implicated in lymphoid tissue function by recruiting either lymphocytes or dendritic cells through CCR6. Our findings corroborate a previous report by Vega et al., which also described an enhancement of CCL2 gene expression in human TNF-α-treated FRCs (10). In contrast, Malhotra et al. reported unaltered expression of CCL2 or CCL20 in mice-derived FRCs and DNCs after LPS *in vivo* stimulation (8).

We observed a more intense migration of monocyte-derived DCs toward TNF-α + IL-1β-treated FRCs. Monocyte-derived DCs expressed CCR2, CCR5, and CCR6 receptors, which are counterparts of CCL2, CCL5, CCL13, and CCL20, genes that were upregulated in treated FRCs. Despite the fact that around 90% of iDCs expressed CCR2 and only around 25% expressed CCR6, both receptors seem to play a role in recruiting iDCs since the independent neutralization of CCL2 or CCL20, their respective ligands, reduced migration toward TNF-α + IL-1β-treated FRCs. There are reports showing that CCR6 varies between individuals and that monocyte-derived iDCs expressing CCR6 are responsive to CCL20 (31, 32). This result confirmed the participation of FRCs in iDCs recruitment through the axis CCR2/CCL2 and CCR6/CCL20. In agreement, a report that correlates chemokines and DCs in sites of oral inflammation, verified that the expression of CCL2 and CCL20 was positively correlated with increased densities of immature CD1a (+) dendritic cells (33). In agreement with our observations, a study using monocyte-derived macrophages also demonstrated a role of the CCR2/CCL2 axis in the migration of these cells (34). The study further demonstrates a role for CCL2 in other leukocytes functions besides chemotaxis (34).

Studies using murine models reported CCL19 secretion by freshly isolated FRCs, both at resting state and after inflammatory stimuli (8, 12), as well as low levels of CCL19 and CCL21 transcripts from cultured FRCs (35). In contrast, our results did not detect altered gene expression of CCL19 or CCL21, and we were not able to detect secretion of CCL19 by resting human FRCs and DNCs or following inflammatory stimuli. Our results are in agreement with Vega et al., who, after TNF treatment, did not detect gene expression of CCL19 or CCL21 by FRCs (10).

CXCL12 *via* CXCR4 is involved in naïve T cell and DCs entry in the LN (14). Our results showed a high expression of CXCR4 in LN-derived lymphocytes. We also showed that CXCL12 secretion was present in FRCs and DNCs cultures. However, CXCL12 secretion was not altered following inflammatory stimuli, corroborating previous reports (8, 36).

Literature reports that FRCs can secrete interleukins (5, 10). Indeed, FRCs, but also DNCs, stimulated with IFN-γ or TNF-α + IL-1β presented altered cytokine expression. Differences in the expression patterns were observed. For instance, FRCs and DNCs treated with TNF-α + IL-1β upregulated IL-6 gene expression, while IL-6-enhanced secretion was only significant for DNCs. In contrast, after IFN-γ treatment, there was IL-6 gene expression upregulation in DNCs but no significant alteration in protein secretion. A previous study described the upregulation of IL-6 transcripts in FRCs after TNF-α treatment (10). IL-6 is a cytokine that plays a role on CD4 T lymphocyte differentiation favoring a Th17 profile (37). FRCs and DNCs treated with TNF-α + IL-1β also displayed a clear upregulation of IL-8 (CXCL8) gene expression and protein secretion. However, IL-8 was overexpressed in FRCs after IFN-γ treatment, while IL-8 protein secretion remained unaltered (14). Corroborating our findings, a lymphoma study also reported upregulation of IL-8 in FRCs after TNF-α treatment (10). IL-8 plays a role as neutrophils chemoattractant, but since there are no neutrophils in LNs, this chemokine could be related to T cells in this microenvironment. Hess et al. reported an unexpected finding of CXCR1 (IL-8 receptor) on a CD8 T cell subset with high cytotoxic potential and terminally differentiated (38).

IL-4I1 was upregulated only in DNCs following TNF-α + IL-1β treatment, while IL7-R was upregulated only in FRCs. IL-7R expression has been described in stromal cells of secondary lymphoid organs and studies reported that IL-7 signaling through IL-7R modulates IL-7 production, consequently playing a role on T cell homeostasis (15, 39). We also observed low levels of IL-7 gene expression (Tables S1–S4 in Supplementary Material) and secretion by FRCs, which were enhanced after treatment with IFN-γ or TNF-α + IL-1β.

IL-10 is an anti-inflammatory cytokine that participates of Th1, NK cells, and macrophages inhibition during infection (40). Like other studies, we did not detect IL-10 expression or secretion by DNCs or FCRs under steady state or after inflammatory stimuli (6, 8, 26). However, IFN-γ treatment enhanced IL-18 binding protein (IL-18BP) in FRCs. IL-18BP is synthetized mainly by mononuclear cells and binds to IL-18 impairing their ligation with the receptor, downregulating the Th1 profile (41). IL-4-induced gene 1 (IL4I1) is only upregulated in DNCs by both treatments and contributed to T cell receptor unresponsiveness in human T helper 17 cells (42).

Most chemokines and cytokines upregulated in FRCs and DNCs after IFN-γ or TNF-α + IL-1β stimuli may be described as pro-inflammatory, and few have been described as hemostatic or with dual function (43). This result suggests that these cells respond accordingly to the inflammatory stimulus received and that they participate of the immune response by recruiting and stimulating immune cells after initial signals of inflammation/infection.

The diversity of interleukins and chemokines secreted by FRCs and DNCs indicate specific signaling and recruitment of these lymphocytes subsets and DCs. This effect should depend on the inflammatory environment. Taken together, our results suggest that DNCs and FRCs play an important role in the recruitment of immune cells to secondary lymphoid organs, including DC and T cells migration. Further, we have confirmed an important participation of FRCs in DCs recruitment through the axis CCR2/CCL2 and CCR6/CCL20.

## Author Contributions

Performed the experiments: LM, DCP, DP, HA, CA, PS, MA, TA, and FR. Analyzed the data: LM, PS, DCP, and FR. Contributed with patient samples: AM, AC, SB, PS, and LR. Contributed reagents/materials/analysis tools: LM. Conceived and designed the experiments: LM. Contributed to the writing and revising the manuscript: LM and PS.

## Conflict of Interest Statement

The authors declare that the research was conducted in the absence of any commercial or financial relationships that could be construed as a potential conflict of interest.

## Supplementary Material

Figure S1 **Double-negative cell (DNC) and fibroblastic reticular cell (FRC) sorting purity**. **(A)** Stromal cells isolated from lymph nodes were sorted in two populations, **(B,C)** DNCs and, **(D–F)** FRCs according to gp38/PDPN expression. After DNCs and FRCs expansion in culture and before each experiment, gp38/PDPN expression was verified, and when purity was lower than 95%, **(E,F)** cells were resorted.

Figure S2 **Gate strategy for monocytes, dendritic cells, and lymphocytes**. **(A)** Monocytes were gated on FSC vs CD14, followed by CD14 vs CD33, and the double-positive cells were analyzed for CD80, CD86, CD209, and HLA-DR, **(B)** immature and, **(C)** mature dendritic cells were gated on FSC vs CD14, followed by CD14 vs CD33, and the CD14-CD33 + positive cells were analyzed for CD80, CD86, CD209, and HLA-DR, **(D)** Lymphocytes were gated on FSC vs CD3, and the CD3 cells were gated for CD4 and CD8, and CD3, CD3CD4, or CD3CD8 positive cells were analyzed for chemokine receptors, expression here illustrated by CCR6.

Figure S3 **Histopathology of human lymph nodes (LNs)**. **(A)** LN derived from larynx cancer patient LN04, **(B)** LNs derived from liver donor LN16. **(C)** LN derived from diverticulitis LN12 hematoxylin–eosin (HE) immunostaining show the organ integrity LN immunohistochemistry for CD3, CD20, or CD68 show the presence of T lymphocytes (CD3), B lymphocytes (CD20), and macrophages (CD68), respectively (magnification of 10×).
