# Supplementary material for: Human Lymph Node-Derived Fibroblastic and Double-Negative Reticular Cells Alter Their Chemokines and Cytokines Expression Profile Following Inflammatory Stimuli
Source: Front Immunol. 2017 Feb 14;8:141. doi: 10.3389/fimmu.2017.00141 (PMC5307266; doi:10.3389/fimmu.2017.00141)
Supplement: Supplementary file 1 [file table_1.docx]

**Supplementary Tables TI, TII, TII and TIV:**

**Table TI – *Chemokines and interleukins induced by IFN-γ on DNCs (LN04. LN12. LN15 and LN16)***

| **LN15 - FC Breast DNC (up IFN)** | **LN12- FC Intestine DNC (up IFN)** | **LN04- FC Larynx DNC (up IFN)** | **LN16-FC Liver DNC (up IFN)** | **Gene**  **Symbol** |
| --- | --- | --- | --- | --- |
| 8.24 | 7.91 | 23.64 | 10.82 | **CCL2** |
| 3.05 | 3.04 | 49.12 | 8.49 | **CCL5** |
| 8.89 | 26.61 | 24.86 | 60.66 | **CCL7** |
| 83.49 | 287.00 | 10.94 | 56.94 | **CCL8** |
| **1.94** | **1.46** | 22.30 | 4.03 | **CCL11** |
| **1.32** | **1.01** | 90.07 | 2.71 | **CCL20** |
| 34.52 | 77.22 | 23.00 | 155.59 | **CCL13** |
| **1.93** | **-1.31** | 122.0 | 3.21 | **CXCL1** |
| 2.72 | 3.40 | 196.41 | 5.22 | **CXCL2** |
| 2.78 | **1.02** | 200.41 | 9.96 | **CXCL8** |
| 192.20 | 155.86 | 28.58 | 388.12 | **CXCL10** |
| 206.27 | 297.37 | 10.37 | 182.47 | **CXCL11** |
| **1.08** | **-1.08** | 70.14 | **1.80** | **IL1A** |
| 3.62 | 4.07 | 17.51 | 7.05 | **IL4I1** |
| 2.86 | **1.91** | 50.20 | 4.29 | **IL6** |
| 4.30 | 6.36 | **1.21** | **1.79** | **IL7** |
| 3.63 | 3.63 | **1.08** | 15.9 | **IL15** |
| 11.10 | 9.36 | 5.48 | 26.95 | **IL32** |
| 8.83 | 2.32 | 11.35 | 2.52 | **IL33** |
| **1.08** | **-1.08** | 70.14 | **1.80** | **IIL1RN** |
| 12.99 | 8.91 | 2.58 | 4.31 | **IL3RA** |
| 6.70 | 6.50 | 2.31 | 11.13 | **IL15RA** |

**(*) We have used colors to represent the gene regulation intensity, genes up-regulated more than 2.0 fold (black), between 1.0 and 2.0 (orange), and genes that were down-regulated (red).**

**Table TII – *Chemokines and interleukins induced by IFN-γ on FRCs (LN04. LN12. LN15 and LN16)***

| **LN15 - FC Breast FRC (up IFN)** | **LN12 - FC Intestine FRC (up IFN)** | **LN04 - FC Larynx FRC (up IFN)** | **LN16 - FC Liver FRC (up IFN)** | **Gene Symbol** |
| --- | --- | --- | --- | --- |
| 6.55 | 7.61 | 3724.64 | 12.37 | **CCL2** |
| 15.37 | 40.38 | 3.42 | 44.73 | **CCL7** |
| 5.14 | 2.21 | 14.9 | **1.65** | **CCL5** |
| 220.35 | 548.88 | 665.79 | 160.32 | **CCL8** |
| 159.37 | 44.43 | 101.95 | 258.92 | **CCL13** |
| **1.59** | 4.5 | 105.97 | 10.71 | **CXCL1** |
| 2.78 | 8.83 | **-1.73** | 6.54 | **CXCL2** |
| 2.13 | 7.05 | 17.86 | 14.59 | **CXCL3** |
| 2.32 | 3.22 | 146.19 | 83.84 | **CXCL8** |
| 446.54 | 476.25 | 1173.02 | 2162.56 | **CXCL9** |
| 1103.64 | 457.31 | 74.76 | 328.52 | **CXCL10** |
| 398.70 | 325.08 | 81.16 | 185.77 | **CXCL11** |
| **-1.02** | **-1.21** | **-102.20** | **-6.38** | **CXCL13** |
| 2.44 | **1.15** | 2.90 | 19.54 | **IL1B** |
| **1.80** | 2.07 | 55.4 | 4.32 | **IL6** |
| **1.49** | 3.19 | **-6.53** | **-2.9** | **IL7** |
| **-1.76** | **-1.58** | 3.36 | 4.22 | **IL11** |
| 3.21 | 3.61 | 138.32 | 189.12 | **IL15** |
| 3.12 | **1.04** | **-1.27** | **-1.14** | **IL17C** |
| **-5.46** | **-1.60** | **-4.81** | **-9.17** | **IL24** |
| 9.95 | 11.13 | **-1.3** | 10.47 | **IL32** |
| 2.89 | 2.71 | 66.58 | 9.29 | **IL33** |
| **1.26** | **1.35** | 16.55 | 7.48 | **IL1RN** |
| **-1.16** | **1.07** | 215.59 | 74.61 | **IL7R** |
| 60.00 | 98.90 | 188.74 | 325.50 | **IL18BP** |
| 7.75 | 8.99 | 3.58 | 12.91 | **IL15RA** |

**(*) We have used colors to represent the gene regulation intensity, genes up-regulated more than 2.0 fold (black), between 1.0 and 2.0 (orange), and genes that were down-regulated (red).**

**Table TIII – *Chemokines and interleukins induced by TNF-α + IL-1β on DNCs (LN04. LN12. LN15 and LN16)***

| **LN15 - FC Breast DNC TNFa IL1 up** | **LN12 - FC Intestine DNC TNFa IL1 up** | **LN04 - FC Larynx DNC TNFa IL1 up** | **LN16 - FC Liver DNC TNFa IL1 up** | **Gene**  **Symbol** |
| --- | --- | --- | --- | --- |
| 45.50 | 16.85 | 8.03 | 36.57 | **CCL2** |
| 9.64 | 221.48 | 2.08 | 2.47 | **CCL3** |
| 73.52 | 260.40 | 4.06 | 59.01 | **CCL5** |
| 17.39 | 27.58 | 21.12 | 88.00 | **CCL7** |
| 15.30 | 21.97 | 134.11 | 2.72 | **CCL8** |
| 2.70 | 3.84 | **1.12** | 24.13 | **CCL11** |
| **1.00** | 14.25 | 164.73 | 7.87 | **CCL13** |
| 244.53 | 639.57 | **-1.5** | 130.96 | **CCL20** |
| 268.01 | 2051.49 | 2.16 | 350.27 | **CXCL1** |
| 524.10 | 828.37 | 3.52 | 505.41 | **CXCL2** |
| 883.90 | 1764.73 | 9.95 | 1401.95 | **CXCL3** |
| 57.54 | 36.67 | 1.05 | 65.5 | **CXCL5** |
| 465.55 | 1138.0 | **1.01** | 602.66 | **CXCL6** |
| 283.91 | 6616.76 | 4.05 | 1662.96 | **CXCL8** |
| 6.83 | 11.38 | 463.06 | 8.49 | **CXCL10** |
| 8.32 | 30.25 | **-1.25** | 77.08 | **IL1A** |
| 1598.84 | 465.99 | 3.75 | 248.94 | **IL1B** |
| 10.20 | 23.15 | 6.85 | 38.23 | **IL4I1** |
| 25.65 | 26.55 | 3.52 | 49.37 | **IL6** |
| **1.28** | 2.24 | 2.13 | 2.00 | **IL7** |
| 30.22 | 18.59 | **-2.16** | 34.77 | **IL11** |
| **1.69** | 2.09 | 2.82 | 3.47 | **IL15** |
| 16.92 | 6.32 | **1.02** | 4.47 | **IL17C** |
| 246.64 | 50.48 | **1.39** | **1.74** | **IL24** |
| 6.45 | 11.35 | 8.43 | 15.10 | **IL32** |
| 53.00 | 17.87 | 2.07 | 16.84 | **IL33** |
| 2.399 | 32.66 | 6.11 | 6.30 | **IL36G** |
| 13.15 | 10.66 | 1.38 | 33.63 | **IL1RN** |
| 2.22 | 2.25 | 5.89 | 3.56 | **IL15RA** |

**(*) We have used colors to represent the gene regulation intensity, genes up-regulated more than 2.0 fold (black), between 1.0 and 2.0 (orange), and genes that were down-regulated (red).**

**Table TIV – *Chemokines and interleukins induced by TNF-α + IL-1β on FRCs (LN04. LN12. LN15 and LN16)***

| **LN15 - FC Breast FRC TNFa IL1 up** | **LN12 - FC Intestine FRC TNFa IL1 up** | **LN04 - FC Larynx FRC TNFa IL1 up** | **LN16 - FC Liver FRC TNFa IL1 up** | **Gene**  **Symbol** |
| --- | --- | --- | --- | --- |
| 23.68 | 15.09 | 12317.06 | 32.99 | **CCL2** |
| 44.20 | 423.76 | 153.98 | 12.33 | **CCL3** |
| 109.41 | 95.99 | 2.11 | 8.90 | **CCL5** |
| 22.57 | 49.23 | 6.53 | 74.15 | **CCL7** |
| 45.83 | 36.35 | 16.80 | **1.49** | **CCL8** |
| 2.44 | 13.86 | 3.63 | 5.98 | **CCL11** |
| 17.97 | 7.77 | 2.47 | 3.08 | **CCL13** |
| 327.70 | 426.21 | 7.35 | 275.17 | **CCL20** |
| 11.61 | 13.70 | 10.32 | 4.40 | **CCL3L3** |
| 64.98 | 594.60 | 5605.64 | 813.41 | **CXCL1** |
| 132.70 | 450.87 | 30.51 | 278.54 | **CXCL2** |
| 226.87 | 1074.94 | 2304.68 | 1799.56 | **CXCL3** |
| 42.24 | 31.71 | 19.8 | 59.07 | **CXCL5** |
| 91.54 | 1390.20 | 277.03 | 1357.49 | **CXCL6** |
| 85.60 | 651.39 | 8310.95 | 10682.34 | **CXCL8** |
| 19.73 | 17.83 | 2.35 | 8.18 | **CXCL10** |
| 4.22 | 2.05 | 4.19 | **1.63** | **CXCL11** |
| 31.66 | 19.35 | 95.25 | 363.93 | **IL1A** |
| 314.46 | 247.43 | 356.60 | 3174.70 | **IL1B** |
| 11.77 | 18.92 | 812.74 | 33.69 | **IL6** |
| **1.14** | **1.76** | 2.58 | **-2.19** | **IL7** |
| 75.00 | 27.15 | 622.71 | 685.03 | **IL11** |
| 2.22 | 2.35 | 31.23 | 83.59 | **IL15** |
| 13.0 | 3.57 | **1.17** | 4.28 | **IL17C** |
| 35.45 | 106.58 | 10.99 | **-3.07** | **IL24** |
| 4.92 | 10.99 | 2.19 | 4.30 | **IL32** |
| 14.37 | 13.07 | 584.91 | 145.03 | **IL33** |
| 13.60 | 44.88 | 42.27 | 14.34 | **IL36G** |
| 10.82 | 16.17 | 1376.31 | 218.45 | **IL1RN** |
| 2.88 | 4.10 | 338.01 | 292.98 | **IL7R** |
| 4.50 | 3.94 | **1.47** | 4.14 | **IL15RA** |

**(*) We have used colors to represent the gene regulation intensity, genes up-regulated more than 2.0 fold (black), between 1.0 and 2.0 (orange), and genes that were down-regulated (red).**
