# Supplementary figures and images for: Human Lymph Node-Derived Fibroblastic and Double-Negative Reticular Cells Alter Their Chemokines and Cytokines Expression Profile Following Inflammatory Stimuli
Source: Front Immunol. 2017 Feb 14;8:141. doi: 10.3389/fimmu.2017.00141 (PMC5307266; doi:10.3389/fimmu.2017.00141)

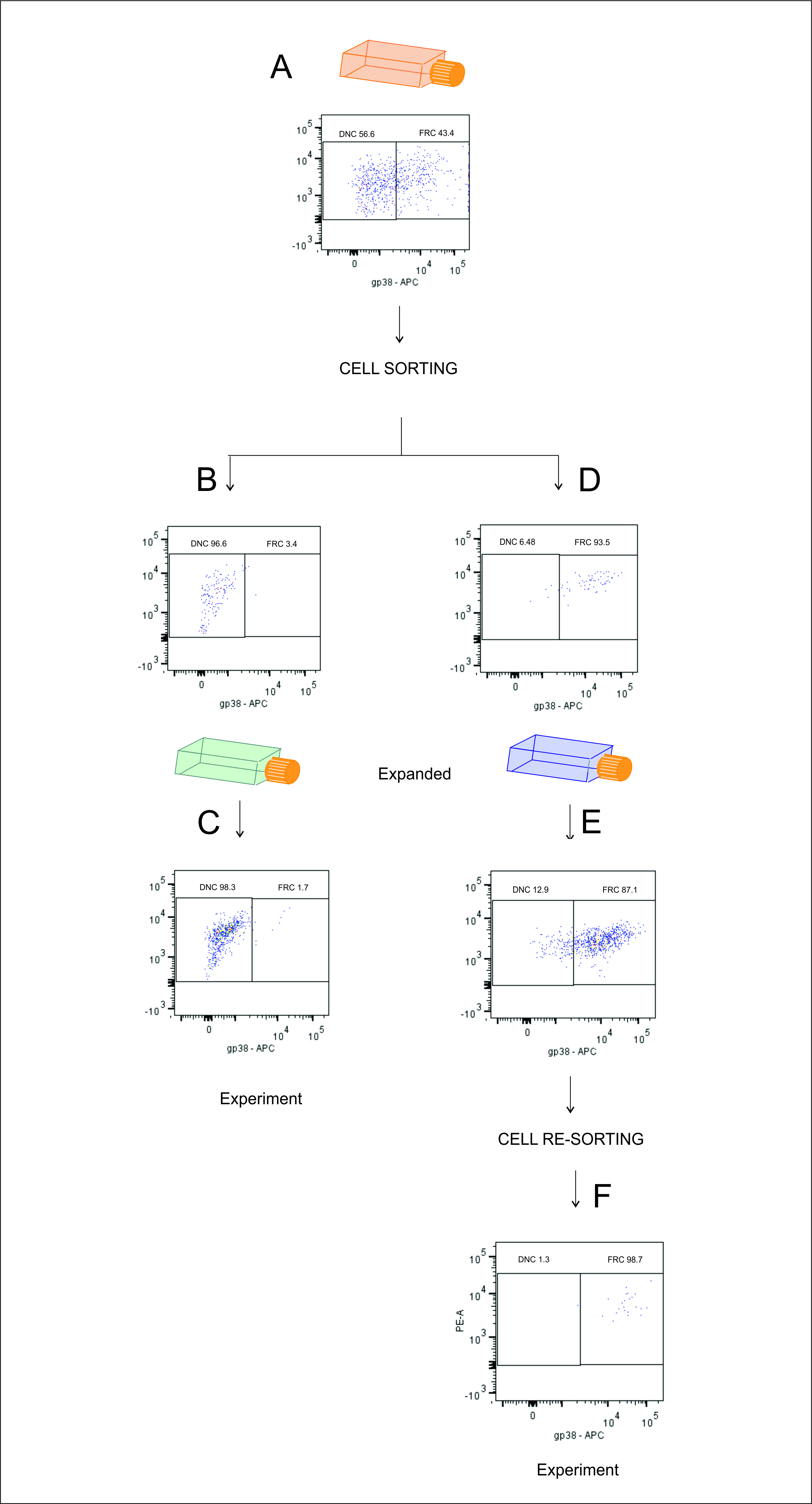

Supplement: Figure S1 — Double-negative cell (DNC) and fibroblastic reticular cell (FRC) sorting purity. (A) Stromal cells isolated from lymph nodes were sorted in two populations, (B,C) DNCs and, (D–F) FRCs according to gp38/PDPN expression. After DNCs and FRCs expansion in culture and before each experiment, gp38/PDPN expression was verified, and when purity was lower than 95%, (E,F) cells were resorted. [file image_1.tif]

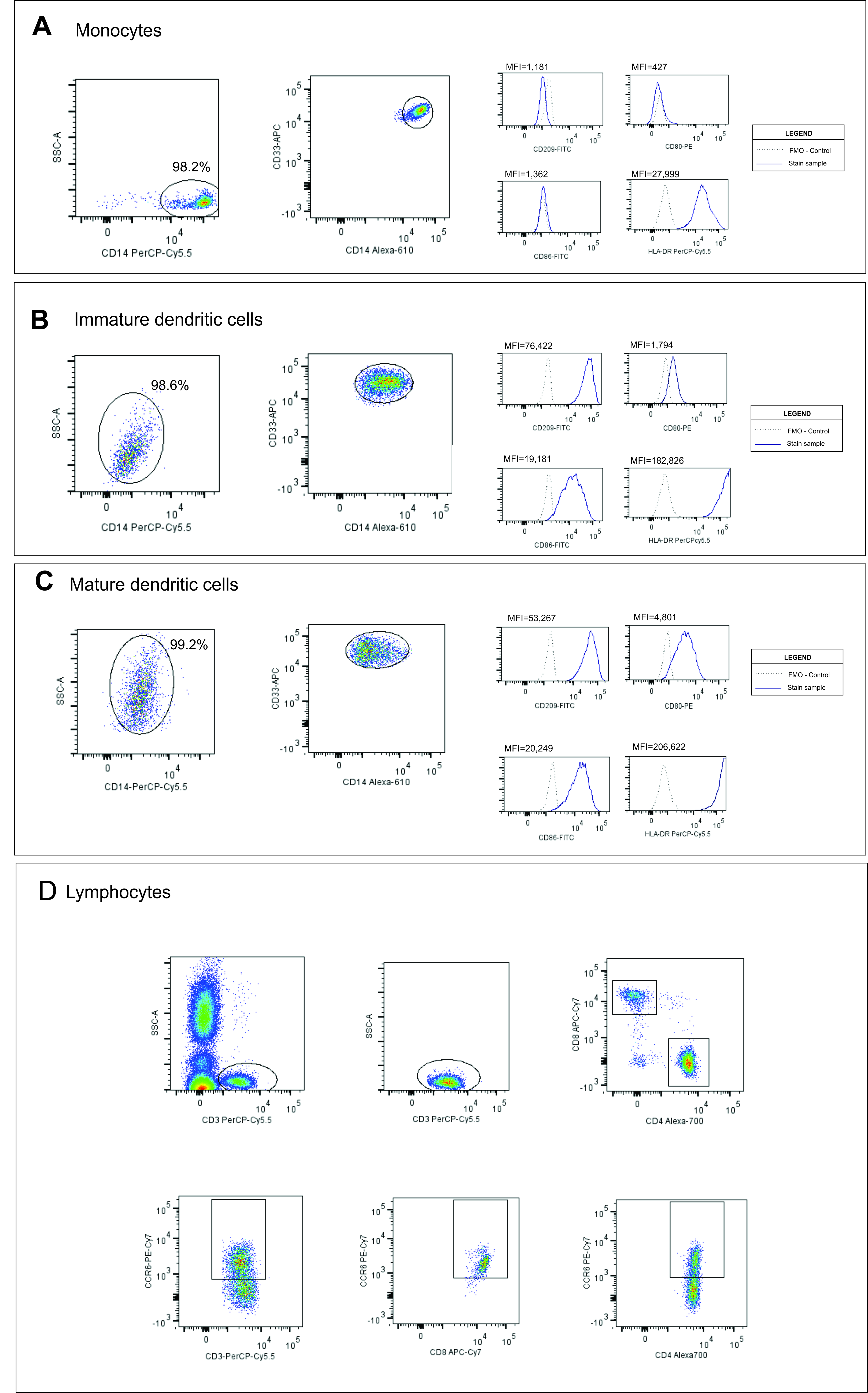

Supplement: Figure S2 — Gate strategy for monocytes, dendritic cells, and lymphocytes. (A) Monocytes were gated on FSC vs CD14, followed by CD14 vs CD33, and the double-positive cells were analyzed for CD80, CD86, CD209, and HLA-DR, (B) immature and, (C) mature dendritic cells were gated on FSC vs CD14, followed by CD14 vs CD33, and the CD14-CD33 + positive cells were analyzed for CD80, CD86, CD209, and HLA-DR, (D) Lymphocytes were gated on FSC vs CD3, and the CD3 cells were gated for CD4 and CD8, and CD3, CD3CD4, or CD3CD8 positive cells were analyzed for chemokine receptors, expression here illustrated by CCR6. [file image_2.tif]

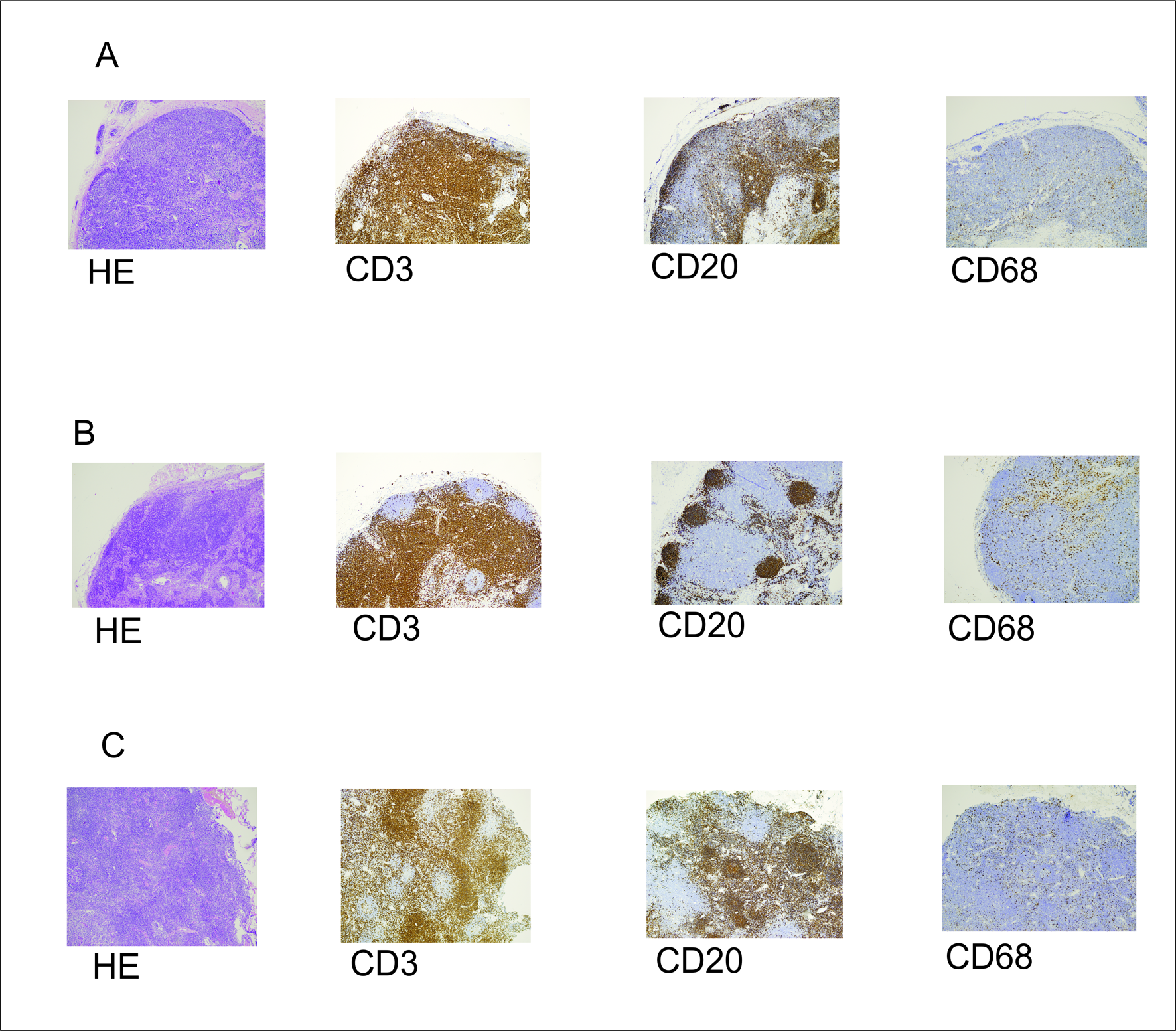

Supplement: Figure S3 — Histopathology of human lymph nodes (LNs). (A) LN derived from larynx cancer patient LN04, (B) LNs derived from liver donor LN16. (C) LN derived from diverticulitis LN12 hematoxylin–eosin (HE) immunostaining show the organ integrity LN immunohistochemistry for CD3, CD20, or CD68 show the presence of T lymphocytes (CD3), B lymphocytes (CD20), and macrophages (CD68), respectively (magnification of 10×). [file image_3.tif]
